# First report on prevalence and risk factors of severe atypical pneumonia in Vietnamese children aged 1–15 years

**DOI:** 10.1186/1471-2458-14-1304

**Published:** 2014-12-18

**Authors:** Phan Le Thanh Huong, Pham Thu Hien, Nguyen Thi Phong Lan, Tran Quang Binh, Dao Minh Tuan, Dang Duc Anh

**Affiliations:** National Institute of Hygiene and Epidemiology, 1 Yersin street, Hanoi, 10.000 Vietnam; National Hospital of Pediatrics, Hanoi, Vietnam

**Keywords:** Pure atypical pathogens, Children, Prevalence, Risk factor, Severe community-acquired pneumonia

## Abstract

**Background:**

Atypical pathogens such as *Mycoplasma pneumoniae*, *Chlamydophila pneumoniae*, and *Legionella pneumophila* are increasingly recognized as important causes of community acquired pneumonia (CAP) worldwide. Such etiological data for Vietnam is scarce and clinical doctors lack accurate information on which to base their diagnosis and treatment of pneumonia. This study identifies the prevalence and risk factors of severe community acquired pneumonia due to these atypical pathogens (severe-*Ap*CAP) in children aged 1–15 years with CAP in a pediatric hospital in Hanoi, Vietnam.

**Methods:**

722 hospitalized children with CAP were recruited for detecting those atypical pathogens, using multiplex PCR and ELISA. Clinical and epidemiological data were collected. Multivariate logistic-regression analyses were performed to evaluate the associations of potential risk factors with severe-*Ap*CAP.

**Results:**

Among 215 atypical pathogen-positive CAP cases, 45.12% (97/215) were severe-*Ap*CAP. Among the severe-*Ap*CAP group, 55.67% (54/97) cases were caused by pure atypical pathogens and 44.33% (43/97) resulted from a co-infection with typical respiratory pathogens. *M. pneumoniae* was the most common, with 86.6% cases (84/97) in the severe-*Ap*CAP group, whereas *C. pneumoniae* and *L. pneumophila* were less frequent (6.19% and 7.22%, respectively). The highest rate of severe-*Ap*CAP was in children younger than two years (65.98%). The differences related to age are statistically significant (*P* = 0.008).

The factors significantly associated with severe-*Ap*CAP were age (OR = 0.84, 95% CI = 0.75-0.93, *P* = 0.001), co-infection with typical bacteria (OR = 4.86, 95% CI = 2.17-10.9, *P* < 0.0001), co-infection with respiratory viruses (OR = 4.36, 95% CI = 1.46-13.0, *P* = 0.008), respiratory/cardiac system malformation (OR = 14.8, 95% CI = 1.12 -196, *P* = 0.041) and neonatal pneumonia (OR = 11.1, 95% CI = 1.06 -116, *P* = 0.044).

**Conclusions:**

Severe-*Ap*CAP presented at a significant rate in Vietnamese children. More than 50% of severe-*Ap*CAP cases were associated with pure atypical pathogen infection. *M. pneumoniae* appeared most frequently. The highest rate of severe-*Ap*CAP was in children younger than two years. Younger age and co-infection with typical bacteria or viruses were the most significant risk factors, while respiratory/cardiac system malformation and neonatal pneumonia were additional potential risk factors, associated with severe-*Ap*CAP in Vietnamese children.

**Electronic supplementary material:**

The online version of this article (doi:10.1186/1471-2458-14-1304) contains supplementary material, which is available to authorized users.

## Background

Worldwide, pneumonia is a leading cause of child death, killing 6.6 million children under the age of five years in 2012 [1]. In Vietnam, community-based studies suggest that every child younger than five years suffers 5 to 8 episodes of acute respiratory infections annually [2], while hospital-based investigations indicate that 35-50% of all pediatric patients are hospitalized due to pneumonia. The Vietnamese Ministry of Health has reported that each year 4000 children younger than 5 years die from pneumonia in Vietnam [3].

Atypical pathogens including *M. pneumoniae*, *C. pneumoniae,* and *L. pneumophila* cause mild, moderate or severe acute respiratory tract infections in children. These pathogens are increasingly recognized as important causes of pneumonia in many countries, but their role in Vietnam has not been well documented. This could diminish the effectiveness of the general guidelines for treatment of acute respiratory tract infections in Vietnamese children. Prior to 2012, only antibiotics of beta-lactam group which do not naturally affect those atypical pathogens were included in the guidelines for pneumonia treatment in children, notably omitting the macrolide group (erythromycin, clarithromycin, azithromycin), which is the first-choice therapeutic agent against atypical pathogen infections in both children and adults. Such incorrect treatment could result in serious pneumonia requiring hospitalization.

Investigations of typical pathogens causing severe pneumonia in children have not included atypical pathogens because it is difficult and not relevant for clinical treatment to detect them by culture methods. This unique study used multiplex polymerase chain reaction (PCR) as the principal method and enzyme-linked immunosorbent assay (ELISA)-based specific IgM antibody for determination of these three atypical pathogens aimed at investigating prevalence of and risk factors for severe community-acquired pneumonia (CAP) caused by *M. pneumoniae, C. pneumoniae* and *L. pneumophila* in hospitalized children with CAP aged 1–15 years old in Vietnam.

## Methods

### Study population

Seven hundred twenty-two hospitalized children aged 1 to 15 years with community-acquired pneumonia were recruited for the prospective hospital-based study at the National Hospital of Pediatrics (NHP), Hanoi. This study was conducted from July 2010 through March 2012, with the approval of Research Ethics Committee, NHP, Hanoi, Vietnam and the agreement from the parents of these patients to participate in the study.

#### The definition of atypical pathogen - positive CAP

(*Ap*CAP) case we used was the following: 1) patients with clinical symptoms of pneumonia, confirmed by radiography and 2) presence of *M. pneumoniae* and/or *C. pneumoniae* and/or *L. pneumophila,* detected in broncho-alveolar lavages, identified by multiplex PCR and ELISA-based specific IgM antibodies against *M. pneumoniae,* or *C. pneumoniae,* or *L. pneumophila* in one of paired sera.

#### Criteria for classification of severe pneumonia in children

Was according to *Pediatric Infectious Diseases Society (PIDS) and the Infectious Diseases Society (IDS) of America* [4]:

+ Major criteria (≥1 major criteria): invasive mechanical ventilation; fluid refractory shock; hypoxemia requiring fraction of inspired oxygen (FiO_2_) greater than inspired concentration or flow feasible in general care area.

+ Minor criteria (≥2 minor criteria): respiratory rate higher than WHO classification for age; apnoea; increased labored breathing; the ratio between partial pressure of arterial oxygen (PaO_2_) and fraction of inspired oxygen (FiO_2_) < 250; multilobar infiltrates (≥2 lobes); Pediatric Early Warning Signs (PEWS) score > 6; altered mental status; hypotension; presence of effusion.

#### Sample size

The sample size of 710 was calculated (Additional file 1) to estimate the prevalence of *Ap*CAP of 23.5% within 0.032 with 95% confidence interval, considering the following parameters: α = 0.05, β = 0.2, and nonresponsive rate = 5% [5].

#### Data collection

At the time of admission, systematic recordings were made for each patient, including medical history, the underlying respiratory symptoms and physical examination. Epidemiological information was collected by interviewing each patient’s parent using a standardized questionnaire.

#### Chest X-ray

After a complete physical examination, chest X-rays were taken. Two senior radiologists read the X-rays and agreed on the conclusion.

#### Microbiological diagnosis

Enrolled patients were investigated for microbiological diagnosis based on respiratory specimens (broncho-alveolar lavages) and two serum samples collected at admission and again after three weeks.

The laboratory tests were performed on blood specimens taken to count leukocytes (WBC), C-reactive protein (CRP) and for the detection of IgM antibodies against *M. pneumoniae*, *C. pneumoniae* and/or *L. pneumophila.*

Broncho-alveolar lavages were used for detection of *M. pneumoniae*, *C. pneumoniae* and *L. pneumophila* specific DNA by multiplex PCR.

In addition, real-time polymerase chain reaction (RT-PCR) was applied to determine the presence of viral respiratory pathogen co-infection (adenovirus, respiratory syncytial virus (RSV), rhinovirus, influenza A & B, parainfluenza 1–3 viruses) using the method of Robin Brittain-Long et al. [6]. For viral RNA extraction we used QIAamp Viral RNA Mini kit (QIAGEN Strasse 1, 40724 Hilden, Germany, Cat No. 52906). The RT-PCR was done using Kit SuperScript III One-Step Kit (Invitrogen Co., 3175 Staley Road, Grand Island, NY 14072, USA). We used the quantitative culture method to detect typical bacterial respiratory pathogens such as *Haemophilus influenzae, Streptococcus pneumoniae,* and *Moraxella catarrhalis* in bronchoalveolar lavages and the results were analyzed according to the published method [7].

### Detection of atypical pneumonia pathogens

#### Multiplex polymerase chain reaction

Broncho-alveolar lavages were kept at −70°C. At test time, all sample volumes were thawed, mixed well, and centrifuged at 15,000 rpm for 10 min at room temperature. Most of the supernatant was discarded and 200 μl of the pellets was used for DNA extraction with QIAamp DNA Mini Kit (QIAGEN Strasse 1, 40724 Hilden, Germany, cat #51304) according to the manufacturer’s instructions.

*M. pneumoniae*, *C. pneumoniae* and *L. pneumophila* DNA were detected by the Multiplex PCR method with primer sets for amplification of the *P1* gene for *M. pneumoniae* (MP-F*:* 5’- AACTATGTTGGTGTATGACCAGTAC-3’ and MP-R: 5’- ACCTTGACTGGAGGCCGTTA-3’) [8]; the *major outer membrane protein gene* for *C. pneumonia* (CP-F:5’-GTTGTTCATGAAGGCCTACT-3’ and CP-R: 5’-CGTGTCGTCCAGCCATTTTA-3’) [9] and *macrophage infectivity potentiator* gene for *L. pneumophila* (mip F2: 5’- GCATTGGTGCCGATTTGG-3’ and mip R2: 5’- GCTTTGCCA TCAAATCTTTCTGAA-3’) [10]. The details of the multiplex PCR applied to detect atypical pneumonia pathogens are presented in Additional file 2.

#### Enzyme-linked immunosorbent assay (ELISA)

Two serum samples were collected from each patient, the first on admission and the second after 3 weeks, and stored at – 20°C until testing. Titers of specific IgM were evaluated using commercial test kits for *M. pneumoniae* ELISA-IgM (*Mycoplasma pneumoniae* ELISA IgM Ref # M1002), *C. pneumoniae* ELISA-IgM (*Chlamydophila pneumoniae* ELISA IgM Ref # M1007) and *L. pneumophila* ELISA-IgM (*Legionella pneumophila* ELISA IgM Ref # M1000) Kits (Vircell S.L, 18016 Granada, Spain), respectively, following the manufacturer’s instructions. The result was recorded as positive when the IgM antibody (Ab) index was greater than 11, as equivocal between 9 and 11, and as negative if under 9.

### Statistical analysis

Frequencies of categorical variables were compared by Fisher’s exact test when appropriate. Binary logistic regression analysis was used to assess factors potentially associated with severe *Ap*CAP due to *M. pneumoniae, C. pneumoniae* and *L. pneumophila*. Multivariate logistic-regression analyses with backward stepwise method were performed to test several models for the associations of severe *Ap*CAP with the potential risk factors. Here, the data are presented as odds ratios with 95% confidence intervals (95% CI). The level of significance was set to 0.05 for all analyses. The above statistical procedures were performed using SPSS version 16.0 (SPSS, Chicago, USA).

## Results

### Characteristics of the study subjects

The socio-demographic characteristics of 215 children suffered from severe or non-severe *Ap*CAP are shown in Additional file 3. Except for age, there were no significant differences between severe-*Ap*CAP and non-severe *Ap*CAP patients with regard to gender, area of residence, kindergarten attendance, living conditions (air-conditioning, dust and/or smoke pollution), or mother’s education level and occupation.

### Prevalence of severe community-acquired pneumonia due to *M. pneumoniae*, *C. pneumoniae and L. pneumophila*in children

As tabulated in Table 1: on the basis of PCR and serological tests, out of 722 hospitalized children with pneumonia, 215 (29.78%) cases were positive for atypical pathogens. The total with *M. pneumoniae* pneumonia was 190/215 cases (88.37%). Among these, *M. pneumoniae* was detected by PCR in 181/190 (95.26%), by ELISA-IgM in 148/190 cases (77.89%) and by both PCR and ELISA in 139/190 (73.16%). *C. pneumoniae* was detected 13/13 cases by PCR, 6/13 (46.15%) by ELISA. *L. pneumophila* was detected in 12/12 cases by PCR and in 11/12 cases by ELISA.

**Table 1:** **Proportion of patients with atypical pathogen positive community - acquired pneumonia on the basis of PCR and serological findings**

Total number of enrolled patients with community acquired pneumonia: 722				
Total number of atypical pathogen positive patients: 215 (29.78%)				
Detected Pathogen	By only ELISA	By only PCR	By both PCR and ELISA	Total of atypical pathogen positive cases
*M. pneumoniae*	9	42	139	190
*C. pneumoniae*	0	7	6	13
*L. pneumophila*	0	1	11	12

Pursuant to the criteria of PIDS and the IDS of America for Classification of Severe pneumonia in Children, of the 215 *Ap*CAP cases, 97 (45.12%) were assigned to the severe-*Ap*CAP group, while 118 (54.88%) were classified as non-severe *Ap*CAP. In both pneumonia forms, *M. pneumoniae* was associated with the highest proportions, for example, 86.60% of severe- *Ap*CAP cases (84/97) and 89.83% (106/118) of non-severe *Ap*CAP. Two remaining atypical pathogens appeared less frequently. *C. pneumoniae* was detected in 6.19% (6/97) of severe-*Ap*CAP and 5.93% (7/118) in non-severe *Ap*CAP while *L. pneumophila* was found in 7.22% (7/97) of severe and 4.24% (5/118) of non-severe cases (Table 2).

**Table 2:** **Prevalence of severe community acquired pneumonia caused by atypical pathogens (N = 215)**

Atypical pathogens causing pneumonia	Total of positive atypical pneumonia cases (%)	Severe- ***Ap***CAP	Non-severe ***Ap***CAP	***P***-value
Total	215 (100)	97 (45.12)	118 (54.88)	
*M. pneumoniae*	190 (81.4)	84 (86.60)	106 (89.83)	
*C. pneumoniae*	13 (6.05)	6 (6.19)	7 (5.93)	0.641
*L. pneumophila*	12 (5.58)	7 (7.22)	5 (4.24)	

Data are number (%). *P*-value by Fisher’s exact test.

In Table 3, it can be seen that among 97 severe-*Ap*CAP cases, ‘pure atypical pathogen’ was associated with 55.67% (54/97) of cases, but only one atypical pathogen was responsible for most of those (51.55%, 50/97). A mixed atypical pathogen infection was present in 4.12% (4/97). The rates of co-infection with other pathogens (typical bacterial pathogens, respiratory viruses, and with three types of pathogen - atypical pathogen, typical bacterial pathogen and respiratory virus in the same sample) in severe-*Ap*CAP group were 27.83% (27/97), 13.4% (13/97) and 3.1% (3/97), respectively. In the non-severe *Ap*CAP group, the rates were 9.3% (11/118); 5.1% (6/118) and 0.8% (1/118) (*P* < 0.0001 and *P* < 0.008), respectively. *Streptococcus pneumoniae* was the most commonly found pathogen in co-infection cases in the severe-*Ap*CAP group (14/27). Co-infection with typical bacteria or a respiratory virus increased the risk of suffering severe pneumonia (OR = 4.62; 95% CI = 2.11-10.1; *P* < 0.0001 and OR = 4.07; 95% CI = 1.46-11.4; *P* = 0.007, respectively) (Table 3).

**Table 3:** **Distribution of severe - atypical pathogen positive pneumonia according to microbiological status**

Pathogens	Severe ***Ap***CAP N = 97	Non-severe ***Ap***CAP N = 118	OR (95% CI)	***P-***value
***Pure atypical pathogen***				
*- Only one atypical pathogen*	50 (51.55)	94 (79.66)	1	-
- Atyp*ical pathogen mix- infection*	4 (4.12)	6 (5.08)	1.25 (0.34-4.65)	0.736
***Co-infection with typically bacterial pathogen***	27 (27.83)	11 (9.32)	4.62 (2.11-10.1)	<0.0001
- *Streptococcus pneumoniae*	14	4		
- *Haemophilus influenzae*	8	4		
- *Moraxella catarrhalis*	1	3		
- *Others (S. aureus; K. pneumoniae…)*	4	0		
***Co-infection with respiratory virus***	13 (13.4)	6 (5.1)	4.07 (1.46-11.4)	0.007
*- RSV*	2	0		
*- Influenza A, B virus*	3	0		
*- Adenovirus*	4	0		
*- Rhinovirus*	4	4		
*- Other virus*	0	2		
***Co-infection with typical bacteria & respiratory virus***	3 (3.1)	1 (0.8)	5.64 (0.57-55.6)	0.139

*P*-value obtained from logistic regression analysis.

Table 4 shows that of the 97 severe-*Ap*CAP cases, 66% (64/97) occurred in children younger than 2 years, 21.65% (21/97) in children from 2 to less than 5 years old, 11.34% (11/97) in children from 5 to less than 10 years, and 1% (1/97) in children 10 or older. The differences related to age were statistically significant (*P* = 0.008).

**Table 4:** **Distribution of severe-ApCAP cases by age**

Age	Severe ***Ap***CAP N = 97 (%)	Non-severe ***Ap***CAP N = 118 (%)
1 **-** < 2 years	64 (65.98)	56 (47.46)
2 **-** < 5 years	21 (21.65)	26 (22.03)
5 **-** < 10 years	11 (11.34)	28 (23.73)
≥10 years	1 (1.03)	8 (6.78)

*P* = 0.008, Fisher exact test.

**Figure 1:**
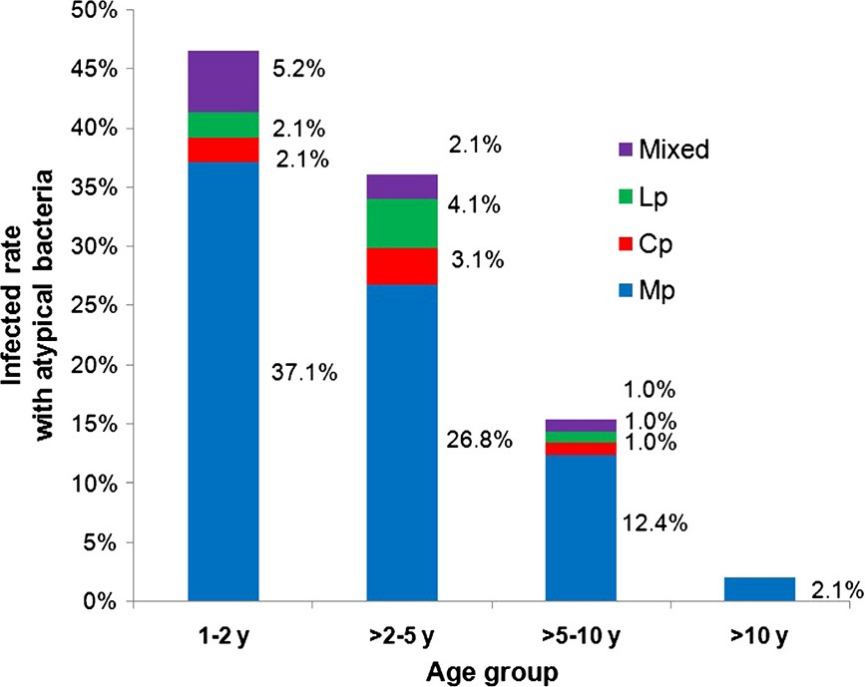
**Distribution of severe-ApCAP cases by ages and by pathogens.** Blue, red, green, and purple indicate the percentage of M. pneumoniae (Blue square Mp), C. pneumoniae (Red square Cp), L. pneumophila (Green square Lp), and atypical pathogen mix-infection (Violet square Mixed), respectively, detected in each age group.

Figure 1 illustrates the role of each atypical pathogen causing severe-*Ap*CAP in children and their distribution by age. *M. pneumoniae* was the most frequently occurring agent, with the highest proportion of infections in children younger than 2 years and decreasing gradually in frequency in older children.

### Risk factors of severe community acquired pneumonia due to atypical pathogens in children

In Table 5, significantly associated risk factors were: age – the younger the child, the greater the chance of severe *Ap*CAP (OR = 0.84; 95% CI = 0.75-0.93; *P* = 0.001), co-infection with typical bacterial pathogens (OR = 4.86, 95% CI = 2.17-10.9, *P* < 0.0001), co-infection with respiratory viruses (OR = 4.36, 95% CI = 1.46-13.0, *P* = 0.008), respiratory/cardiac system malformation (OR = 14.8; 95% CI = 1.12 -196; *P* = 0.041) and neonatal pneumonia (OR = 11.1; 95% CI = 1.06 -116; *P* = 0.044). None of the other variables investigated, including gender, nutrition status, weight at birth, history of respiratory/cardiac system disease, asthma, duration of illness prior to hospitalization, antibiotic usage, and others were significantly associated with severe-*Ap*CAP (data not shown).

**Table 5:** **Associated factors of severe - ApCAP in multiple logistic regression analysis with backward stepwise method**

Variables		OR (95% CI)	***P*** − value
Age	Year	0.84 (0.75-0.93)	0.001
Co-infection status	One atypical pathogen	1.0	-
	Mixed atypical pathogens	0.96 (0.22-4.25)	0.959
	Atypical pathogen + typical bacteria	4.86 (2.17-10.9)	<0.0001
	Atypical pathogen + respiratory virus	4.36 (1.46-13.0)	0.008
	Atypical pathogen + typical bacteria + respiratory virus	5.30 (0.53-52.8)	0.155
Resp/cardiac system malformation	No	1.0	-
	Yes	14.8 (1.12-196)	0.041
Neonatal pneumonia	No	1.0	-
	Yes	11.1 (1.06-116)	0.044

Odds ratio (OR) and *P-*values were adjusted by all variables in the table.

## Discussion

### The role of atypical pathogens in severe community-acquired pneumonia in children

Worldwide, these three atypical pneumonia agents are each responsible for 10% to 30% of CAP in children [11]. Among them, *M. pneumonia* is the most common causative agent, followed by *C. pneumoniae* and *L. pneumophila*. This is the first investigation of severe *Ap*CAP in children in Vietnam based on molecular and serological diagnosis.

Pursuant to the criteria of PIDS and the IDS of America for severe pneumonia, the 215 *Ap*CAP cases in our study were classified as severe-*Ap*CAP (45.12%) or non-severe *Ap*CAP (54.88%). In both groups, *M. pneumoniae* accounted for the majority of infections (86.6% and 89.83%), while *C. pneumoniae* and *L. pneumophila* were much less common (<10% for each).

According to the literature, atypical pathogens, especially *M. pneumoniae* and *C. pneumonia,* could be considered as co-infective agents in severe pneumonia [12–15], significantly enhancing the severity of the pneumonia. However, in our study, pure atypical pathogen infections played an important role in severe-*Ap*CAP, causing more than half of severe-*Ap*CAP cases. Co-infection with other pathogens (typical bacterial pathogens or viruses) was found in just under half of the cases. Among these, more than 50% of co-infection with typical bacteria was with *Streptococcus pneumoniae*.

A number of published reports [16–20] suggest that *M. pneumoniae* and *C. pneumoniae* infections occur more frequently in children older than two years and in school-age children (older than five years). In our study population, the highest proportion of general *Ap*CAP and of particularly severe-*Ap*CAP cases occurred in children younger than two years. These differences by ages were statistically significant. In addition, *M. pneumoniae* appeared to be the most important atypical pathogen in all age groups with severe-*Ap*CAP, compared to other atypical pathogens, especially in children younger than two years. Hence, it can be said that pure atypical pathogen infections, especially with *M. pneumoniae* appear to play an important role in pneumonia/severe pneumonia in young children in Vietnam.

Before this study, based on published data outside of Vietnam [20–23], most Vietnamese pediatricians omitted atypical pathogens, especially *M. pneumoniae* (causing pneumonia) in children younger than 2 or 5 years in their diagnosis and treatment. *This study provides evidence for the need to revise the existing diagnostic and treatment criteria in Vietnam*.

### Risk factors related to severe community-acquired pneumonia due to atypical pathogens in children

The results above strongly suggest that pure atypical pathogen infections play an important role in causing severe-*Ap*CAP in Vietnamese children. Using appropriate statistical methods, we investigated potential risk factors for these infections. The results identified several significant risk factors associated with severe-*Ap*CAP: age, co-infection status, respiratory/cardiac system malformation, and neonatal pneumonia.

Age is generally considered as a factor associated with incidence of pneumonia and mortality rate in children younger than two years. A Thai study reported that the mean age of severe *M. pneumoniae* CAP was around 21 months, in severe *C. pneumoniae* CAP about 49 months, and again about 24 months in severe co-infection pneumonia [24]. However, there are reports of severe-*Ap*CAP occurring in healthy adolescents and adults [25, 26].

In our study, the highest proportion of severe-*Ap*CAP infections occurred in children younger than two years; our analysis also indicated that age is significantly associated with severe-*Ap*CAP: the younger the child, the more likely that the *Ap*CAP will be severe.

In cases of co-infection in our study, it is difficult to identify which pathogen was the first cause, especially because the National Hospital of Pediatrics in the capital city-Hanoi is often the last in a line of many health care providers. At the time the patient was admitted, laboratory tests such as WBC, CRP often indicate an acute bacterial infection. However, it is probable that when more microbiological pathogens afflict the patient, the resulting illness will be more severe [13, 15].

In our study, the proportion of severe pneumonia due to pure atypical pathogens was considerable, more than half. Co-infection with typical bacteria was also significantly associated with severe *Ap*CAP. Co-infection between *M. pneumoniae* and *S. pneumoniae* appeared most frequently, more than half of the co-infections, followed by *H. influenzae* and then others (*Moraxella catarrhalis*, *Klebsiella pneumoniae*, *Staphylococcus aureus*). Co-infections with respiratory viruses (*adenovirus*, *rhinovirus*, *influenza* A, B viruses and RSV) could also enhance the severity of pneumonia. The contribution of co-infections to the severity of atypical pneumonia was assessed by multi-variable logistic-regression analysis. Co-infection with one of the typical bacterial pathogens could increase severe-*Ap*CAP cases by 4.86 times and with respiratory viruses to 4.36 times compared with pneumonia caused by only one atypical pathogen.

Atypical pathogen co-infection was not significantly associated with severity when compared with CAP due to only one atypical pathogen. Co-infection with typical bacterial pathogen and respiratory virus was also not significantly associated with severe *Ap*CAP. This was somewhat unexpected and may be due to the small number of such samples.

Health status is one factor that could influence recovery from pneumonia or severity of the illness in children. In our study, respiratory/cardiac system malformations and neonatal pneumonia were identified as potential risk factors in severe *Ap*CAP. These factors have a physical correspondence with recurrent bronchitis or pneumonitis in childhood [27, 28]. However, the vibration amplitude of both variables was rather large, so the study should be expanded to confirm this result.

#### Limitations of the study

In general, atypical bacteria, especially *M. pneumoniae* and *C. pneumoniae* are difficult to isolate. Serodiagnostic methods based on IgM detection or elevated IgG titer depend on the duration of the illness and on the immune response of each individual. The development of PCR has provided an alternative diagnostic method for etiological agents that are difficult to culture or detect. PCR-based assays can provide results quickly and avoid the risk of false negative results in conventional culture methods.

In this study, we used a combination of conventional multiplex PCR and specific IgM antibody determination to detect three targeted atypical pathogens (or atypical bacteria) and to maximize the etiological diagnosis. However, a limitation was that we could not use real-time PCR for detection of the atypical bacteria, which is much more sensitive than conventional PCR. Due to financial limitations, we could not expand to use other diagnostic tools such as antigen detection test in urine for *Legionella* infection or real-time PCR for detection of typical bacterial infections in patients who had taken antibiotics prior to admission.

## Conclusions

In Vietnamese children, a significant proportion of severe community-acquired pneumonia was caused by the atypical pathogens (*M. pneumoniae, C. pneumoniae* and *L. pneumophila). Mycoplasma pneumoniae* was the most common pathogen in severe-*Ap*CAP.

Among severe-*Ap*CAP cases, pure infections with atypical pathogens accounted for more than half of the cases. The highest rate of severe-*Ap*CAP cases was seen in children younger than two years. Several risk factors were identified, among which age and co-infection with typical bacterial pathogens or respiratory viruses were the most significant. Respiratory/cardiac system malformation and neonatal pneumonia were also important risk factors for severe-*Ap*CAP in Vietnamese children.

Therefore, these pathogens, especially *M. pneumoniae* should be added to the diagnostic protocols for children with pneumonia, and to surveillance of respiratory infections, especially among younger children.

## Electronic supplementary material

Additional file 1: **Sample size calculation to estimate prevalence of atypical pathogen positive community acquired pneumonia (** ***Ap*** **CAP).** (DOCX 22 KB)

Additional file 2: **Multiplex PCR applied to detect atypical pneumonia pathogens.** (DOCX 24 KB)

Additional file 3: **Socio-demographic characteristics of the children in the study.** (DOCX 23 KB)

## Abbreviations

*Ap*CAP: Atypical pathogen positive community-acquired pneumonia
CAP: Community-acquired pneumonia
ELISA: Enzyme-linked immunosorbent assay
PIDS and IDS of America: Pediatric infectious diseases society and the infectious diseases society of America
PCR: Polymerase chain reaction
RT-PCR: Real-time polymerase chain reaction
severe-*Ap*CAP: Severe form of community-acquired pneumonia due to atypical pathogens.
