# Supplementary material for: First report on prevalence and risk factors of severe atypical pneumonia in Vietnamese children aged 1–15 years
Source: BMC Public Health. 2014 Dec 18;14:1304. doi: 10.1186/1471-2458-14-1304 (PMC4300840; doi:10.1186/1471-2458-14-1304)
Supplement: Supplementary file 1 — Additional file 1: Sample size calculation to estimate prevalence of atypical pathogen positive community acquired pneumonia ( Ap CAP). (DOCX 22 KB) [file 12889_2014_7388_MOESM1_ESM.docx]

**Additional file 1**

***Sample size calculation to estimate prevalence of atypical bacteria positive community-acquired pneumonia (ApCAP)***

The sample size of 710 was calculated to estimate the prevalence of *Ap*CAP of 23.5% within 0.032 with 95% confidence interval, considering the following parameters: α = 0.05 (value of α is the probability of type I error), β = 0.2 (value of β is the probability of type II error, or (1-power) of the test), and non-response rate = 5%, using the following formulation:

$$n=\frac{Z_{1-\alpha/2}^{2}p(1-p)}{d^{2}}$$

- α = 0.05, β=0.2, Z_1-α/2_ = 1.96,
- *p* is estimated prevalence of *Ap*CAP: 0.235, *q* = 1 −*p* = 0.765
- d is acceptable margin of error for proportion being estimated = 0.032,
- Non-responce rate is about 5%.
- Estimated sample size = n + 0.05n ≈ 710 subjects.
