# Supplementary material for: First report on prevalence and risk factors of severe atypical pneumonia in Vietnamese children aged 1–15 years
Source: BMC Public Health. 2014 Dec 18;14:1304. doi: 10.1186/1471-2458-14-1304 (PMC4300840; doi:10.1186/1471-2458-14-1304)
Supplement: Supplementary file 2 — Additional file 2: Multiplex PCR applied to detect atypical pneumonia pathogens. (DOCX 24 KB) [file 12889_2014_7388_MOESM2_ESM.docx]

**Additional file 2: Multiplex PCR applied to detect atypical bacteria**

Broncho-alveolar lavages were kept at -70^0^C until testing. At test time, all samples were thawed, mixed well, and centrifuged at 15.000rpm for 10 min. at room temperature. Most of the supernatant was discarded and 200µl of the pellets were used for DNA extraction with QIAamp DNA Mini Kit (QIAGEN Strasses 1, 40724 Hilden, Germany, Cat No. 51304) according to the manufacturer’s instruction. Molecular detection was performed by an in-house multiplex PCR, which used previously evaluated primer sets for *L. pneumophila* (LP) [1], except for *M. pneumoniae* (MP) and *C. pneumoniae* (CP). We used amplification of *P1* gene for MP *(MP-F:*5’-AACTATGTTGGTGTATGACCAGTAC-3’ and MP-R: 5’-ACCTTGACTGGAGGCCGTTA-3’) and amplification of *outer membrane protein* gene for CP, in which the reverse primers **(**CPTM2r: 5’- CGT GTC GTC CAG CCA TTT TA-3’) were all self-designed by Nagasaki Research Team (Nagasaki University, Nagasaki, Japan) [2], while the forward primer (CPc1 or HL-1: 5’- GTT GTT CAT GAA GGC CTA CT-3’) was obtained from the literature [3]. All of the primers were evaluated for specificity (no nonspecific bands were observed in any of the tests evaluated). In addition, these primer sets have been used in their own PCR to detect MP, CP and LP at the Department of Clinical Medicine, Institute of Tropical Medicine, Nagasaki University, Nagasaki, Japan and published [2]**.**

In our study, based on those primers, we optimized reactive conditions of a conventional multiplex PCR which we have used in this study. After being optimized, this multiplex PCR assay was shown in our laboratory to have a detection limit of ≤ 10 colony forming units per 1 reaction tube for each target bacteria; repeats of the result were stable in five separate tests. Briefly, the reaction mixture for the optimized multiplex PCR was prepared in a total volume of 25 µl as follows: 13µl of QIAGEN Multiplex PCR Kit (QIAGEN, 40724 Hilden, Germany, Cat No. 206145), 1.5 µl of 25mM MgCl_2_ as supplementation (final concentration was 2.25 mM**),** 7.5pmol of each MP primer; 30pmol of each CP primer; 10pmol of each LP primer (amplification of *macrophage infectivity potentiator* gene), 1µl of DMSO (99.5%) and 2 µl of extracted nucleic acid from each sample. The multiplex PCR for these samples was performed using MasterCycler epGradient S **(**Eppendorf AG, 22331 Hamburg, Germany**)** with the following conditions: initial activation of 4 cycles of (96^0^C for 3 min; 55^0^C for 30 s; 72^0^C for 45 s), subsequent to 40 cycles of (94^0^C for 35 s; 55^0^C for 40 s; 72^0^C for 1min), last extension time of 10 min at 72^0^C. Amplification products were analyzed by electrophoresis through a 2.0% combined agarose gel (1.0 g Nusieve GTG agarose mixed with 1.0 g Seakem GTG agarose (LONZA Group Ltd, CH-4002 Basel, Switzerland) in TAE buffer (Invitrogen, 3175 Staley road, Grand Island, NY 14072, USA. Cat No. 15558-042). SYBR® Safe DNA gel stain (Invitrogen, 3175 Staley road, Grand Island, NY 14072, USA) was used to visualize the PCR products. The appearance of 229, 287 and 168 base pair (bp) bands corresponding to MP, CP and LP, respectively was considered positive. In each experiment, negative and positive controls for each bacterium were used. Sample processing, PCR reaction mix preparation, PCR amplification, and analysis of PCR products were carried out in adequately separated rooms. Equivocal PCR results were confirmed by single PCR. No specimen displayed discordant results and most of the patients with positive results showed good outcome with appropriate antibiotic therapy, except for three very severe cases who died (3/97).

**References:**

1. Nagai T, Sobajima, H. Iwasa, M. Tsuzuki, T. Kura, F. Amemura –Maekawa, and Watanabe H:**Neonatal Sudden death due to *Legionella pneumoniae* Associated with Water Birth in a Domestic Spa Bath**. *J ClinMicrobiol* 2003,**41**:2227-2229.

2. Masahiro Takaki, Takahiro Nakama, Masayuki Ishida, Hitomi Morimoto, Yuka Nagasaki, RinaShiramizu, NaohisaHamashige, Masayuki Chikamori, Laymyint Yoshida, KoyaAriyoshi, Motoi Suzuki, and Konosuke Morimoto: **High Incidence of Community-Acquired Pneumonia among Rapidly Aging Population in Japan: a Prospective Hospital-Based Surveillance. *Jpn. J. Infect. Dis.*** 2014. **67**: 269-275

3. Campbell L. A, M Perez Melgosa, D J Hamilton, C CKuo and J T Grayston:**Detection of Chlamydia pneumoniae by polymerase chain reaction**. *J ClinMicrobiol* 1992, **30**(2): 434-439.
