# Supplementary material for: First report on prevalence and risk factors of severe atypical pneumonia in Vietnamese children aged 1–15 years
Source: BMC Public Health. 2014 Dec 18;14:1304. doi: 10.1186/1471-2458-14-1304 (PMC4300840; doi:10.1186/1471-2458-14-1304)
Supplement: Supplementary file 3 — Additional file 3: Socio-demographic characteristics of the children in the study. (DOCX 23 KB) [file 12889_2014_7388_MOESM3_ESM.docx]

**Additional file 3. Socio-demographic characteristics of the children in the study**

| Variables | Severe *Ap*CAP  (N = 97) | Non-severe *Ap*CAP  (N = 118) | P-value* |
| --- | --- | --- | --- |
| Age (months)† | 3.2 (1.7-7.0) | 2.2 (1.2-4.3) | 0.001 |
| Gender |  |  |  |
| Female | 37 (38.1) | 52 (44.1) |  |
| Male | 60 (61.9) | 66 (55.9) | 0.381 |
| Resident area |  |  |  |
| Rural | 46 (47.4) | 41 (34.7) |  |
| Mountain | 12 (12.4) | 11 (9.3) | 0.072 |
| Urban | 39 (40.2) | 66 (55.9) |  |
| Having air conditioning |  |  |  |
| No | 58 (59.8) | 56 (47.5) |  |
| Yes | 39 (40.2) | 62 (52.5) | 0.071 |
| Going to kindergarten |  |  |  |
| No | 39 (40.2) | 38 (32.2) |  |
| Yes | 58 (59.8) | 80 (67.8) | 0.223 |
| Living condition polluted by dust and smoke |  |  |  |
| No | 67 (69.1) | 70 (59.3) |  |
| Yes | 30 (30.9) | 48 (40.7) | 0.140 |
| Mother education level |  |  |  |
| Elementary and intermediate | 31 (32.0) | 33 (28.0) |  |
| Secondary | 42 (43.3) | 48 (40.7) | 0.552 |
| Post secondary | 24 (24.7) | 37 (31.4) |  |
| Mother occupation |  |  |  |
| Unemployed | 22 (22.7) | 27 (22.9) |  |
| Farmer | 31 (32.0) | 30 (25.4) | 0.722 |
| Office staff | 35 (36.1) | 47 (39.8) |  |
| Other | 9 (9.3) | 14 (11.9) |  |

Data are number (%) unless otherwise indicated. †Data are median (interquartile range).

* P-values by chisquare test.
